# Editorial: Emerging indigenous food processing in solving nutrition problems

**DOI:** 10.3389/fnut.2024.1473715

**Published:** 2024-08-26

**Authors:** Fitriyono Ayustaningwarno, Diana Nur Afifah, Yonathan Asikin

**Affiliations:** ^1^Department of Nutrition Science, Faculty of Medicine, Universitas Diponegoro, Semarang, Indonesia; ^2^Center of Nutrition Research, Universitas Diponegoro, Semarang, Indonesia; ^3^Department of Bioscience and Biotechnology, Faculty of Agriculture, University of the Ryukyus, Okinawa, Japan; ^4^United Graduate School of Agricultural Sciences, Kagoshima University, Kagoshima, Japan

**Keywords:** indigenous food, indigenous food processing, local food, food processing, nutrition problems, traditional food

Emerging methods for processing indigenous foods can contribute to addressing nutritional challenges by enhancing the availability and affordability of indigenous foods, boosting their nutritional quality, and maintaining the heritage of customary food practices. Indigenous foods frequently offer greater nutritional benefits compared to non-indigenous alternatives, largely because they are cultivated or prepared using conventional techniques free from synthetic chemicals or pesticides. Furthermore, these foods typically provide a rich source of vitamins, minerals, and antioxidants, which are essential for maintaining good health: for instance, non-centrifugal cane sugars, which are indigenous to many countries, were found high in minerals and bioactive compounds compared to refined sugar (1). However, indigenous foods can be difficult to access and afford, as they are often not widely available in grocery stores: for example, wood apple (*Limonia acidissima* L.), an indigenous fruit from Rembang Regency, Indonesia, despite having high vitamin C and antioxidant activity, it was difficult to find in other places (2). Additionally, traditional methods of processing indigenous foods can be time-consuming and labor-intensive: for example, lindur fruit (*Bruguiera gymnorrhiza* L.), a species of mangrove, has to be soaked in water for 24 h to remove the bitter taste (3), which can make them less appealing to consumers.

Innovative techniques in processing indigenous foods are key to overcoming accessibility and affordability issues related to indigenous foods. Indigenous groups, for instance, are embracing contemporary tools like food dehydrators and solar dryers, enabling them to streamline the production of traditional foods more effectively: for example, vacuum frying, an indigenous fruit processing in Asia that is able to maintain high vitamin C and β-carotene content of its fruits while having a crispy, and appealing colors (4), a process optimization is often also required to achieve the desirable quality attribute: for example, moisture as a function of time and oil temperature. This moisture function further emphasizes texture and fat uptake as a function of moisture. The moisture function also indirectly describes reaction rate as a function of temperature in the context of color (5). Additionally, some indigenous communities are partnering with food companies to create and market indigenous foods to a wider audience (6).

This Research Topic contains a number of manuscripts on the emerging indigenous food processing in solving nutrition problems. Six manuscripts are included: four original research articles, one review, and one mini review. They show various indigenous food materials including ginger, tempe, and parijoto fruit, that can help to solve nutrition problems.

Two manuscripts present the effects of processing on food quality. Astawan et al. compare germinated and non-germinated velvet bean and soybean tempe, focusing on their hardness, firmness, color, antioxidant capacity, and sensory attributes. Findings reveal that germination enhances the qualities of velvet bean tempe, making it a viable option for tempe production, despite some limitations like lower protein content. This research contributes valuable insights into tempe's ingredient diversity, highlighting germination's role in improving tempe's nutritional and sensory characteristics. Ananingsih et al. determined the effect of various tween stabilizers to enhance bioavailability and control the release of parijoto fruit nano emulsion anthocyanins. The optimal nano emulsion was achieved with 12% Tween 80 and a 7.5% fruit extract, showing improved particle size and distribution.

This Research Topic also presents the physicochemical properties of indigenous food. Ayustaningwarno et al. captured the physicochemical properties of ginger. The review highlights ginger's components like gingerol, shogaol, paradol, and zingerone, which contribute to its health properties. Mechanisms such as Nrf2 signaling pathway activation and NF-κB activation are discussed, explaining ginger's effectiveness in modulating the immune system by reducing inflammation and oxidative stress. The review also touches on ginger's historical use as a food and medicine, its role in disease prevention, and future research directions. Collaboration between researchers and industry is advocated to advance ginger applications further and explore its potential to enhance human immunity.

Three manuscripts present an overview of indigenous diets. Awudi et al. find that migrants consumed more unhealthy foods, contributing to higher obesity rates. Key factors linked to obesity among migrants included late-night meals, eating red and processed meats, refined grains, and sweets. With 678 participants mainly aged 18–36, the study underscores the impact of adopting a Western diet and meal timing on obesity among African migrants. It suggests managing dietary choices and eating habits could mitigate obesity risks, highlighting a pronounced need for dietary education and intervention in migrant populations. Mbhatsani et al. constructed a model that emphasizing collaboration across various stakeholders including chefs, dietitians, farmers, and educators. Influenced by WHO's strategies for health promotion, it focuses on medical, educational, behavioral change, empowerment, and societal enhancement to encourage vegetable intake. Heaney et al. identify and explore adaptable technologies, including storage, packaging, drying, canning, pickling, and fermentation, which could optimize conditions to improve food safety and shelf life. It underscores the necessity of integrating these technologies within indigenous food systems to preserve food quality, enhance accessibility, and respect cultural values. It seeks to support the sovereignty and independence of indigenous food systems and contribute to sustaining the indigenous food culture.

In summary, this Research Topic emphasizes the importance of emerging indigenous food processing in solving nutrition problems. This topic is gaining attention. However, some areas were less investigated, such as molecular changes during indigenous food processing and their mechanism to contribute to solving nutrition problems. This information would be critical to provide scientific guidelines for significant indigenous food and food processing in solving nutrition problems.
